# Diagnostic Accuracy of Centor Score for Diagnosis of Group A Streptococcal Pharyngitis among Adults in Primary Care Clinics in Malaysia

**DOI:** 10.21315/mjms2022.29.4.9

**Published:** 2022-08-29

**Authors:** AbdulRahman Muthanna, Nurainul Hana Shamsuddin, Aneesa Abdul Rashid, Sazlina Shariff Ghazali, Rukman Awang Hamat, Maliza Mawardi, Hani Syahida Salim, Siti Zulaikha Zakariah

**Affiliations:** 1 Department of Medical Microbiology, Faculty of Medicine and Health Sciences, Universiti Putra Malaysia, Selangor, Malaysia; 2 Department of Biomedical Sciences, Faculty of Medicine and Health Sciences, Universiti Putra Malaysia, Selangor, Malaysia; 3 Department of Family Medicine, Faculty of Medicine and Health Sciences, Universiti Putra Malaysia, Selangor, Malaysia

**Keywords:** pharyngitis, Centor score, primary care, antibiotics, prescription

## Abstract

**Background:**

Pharyngitis is a common presentation seen in general practice, but it is difficult to differentiate whether its etiology is viral or bacterial. The Centor score gives an approximation of the etiology of the pharyngitis, which informs physicians of the need to prescribe antibiotics. This study aimed to assess the validity of the Centor score in diagnosing Group A streptococcal (GAS) pharyngitis amongst adults in Malaysia.

**Methods:**

This cross-sectional study was conducted to compare the clinical criteria of the Centor score to the gold standard throat swab culture results amongst 215 adults presenting with sore throat in primary care clinics. The participants were adult patients who complained of sore throat and visited the three public primary care clinics in Sepang, Malaysia. The convenience sampling method was used. The throat swabs were analysed for β-haemolytic streptococci. Demographic and clinical data, including the Centor score, were analysed in relation to the pathogen.

**Results:**

Pharyngitis was diagnosed in 130 (60.5%) of the participants. Six isolates (2.4%) were identified as GAS pharyngitis. Both Centor scores 3 and 4 had a sensitivity of 50%, and specificities of 97.6% and 100%, respectively.

**Conclusion:**

A Centor score < 3 is favourable for excluding a diagnosis of GAS pharyngitis. Centor scores 3 and 4 require further examination to confirm a diagnosis of GAS pharyngitis.

## Introduction

Pharyngitis is one of the commonest upper respiratory tract infections (URTIs) seen in primary care clinics (1). In Malaysia, URTI makes up nearly 30% of cases in primary care (2). Viruses mainly cause acute pharyngitis in adults, with only 5%–10% caused by Group A *Streptococcus* (GAS), which is also known as *Streptococcus pyogenes* (1, 3). Bacterial pharyngitis caused by GAS can trigger an autoimmune inflammatory response in the form of acute rheumatic fever and glomerulonephritis weeks after the symptoms resolve (1).

Many physicians tend to prescribe antibiotics for pharyngitis in their clinical practice, although evidence has shown that they are mostly caused by viruses (4). Studies have shown inappropriate antibiotic prescribing practices for URTIs in Malaysian primary care (5). Rates for antibiotic prescribing in URTIs have been shown to be as high as 46.7% in Malaysian primary care settings, which exceeds the expected prevalence of GAS pharyngitis in adults and children (6). Overprescribing of antibiotics has very serious health effects, including severe reactions, promotion of antibiotic resistance and significant additions to the cost of healthcare (7–8).

The gold standard for diagnosing GAS pharyngitis is throat swab culture. However, this usually takes 2 days–3 days for the bacterial growth to be identified (1, 9). Moreover, throat swab culture is not available in most Malaysian primary care clinics. A quick and effective diagnosis of pharyngitis on the first visit is needed, as without treatment, pharyngitis resolves after 3 days–5 days. To prevent the sequelae of GAS pharyngitis, such as rheumatic fever or acute glomerulonephritis, a 10-day course of antibiotics is necessary (9).

In 1981, Centor et al. (10) developed a set of criteria to predict the likelihood of GAS pharyngitis in adult patients. Their study showed that the Centor score correctly predicted residents’ guesses with a specificity of 0.92 and a sensitivity of 0.90. This score could limit the over-prescription of antibiotics and reduce the emergence of antibiotic resistance and lower healthcare costs (10).

This study aims to evaluate the diagnostic use of the Centor criteria for Malaysian primary care clinics by means of sensitivity, specificity, positive predictive value (PPV) and negative predictive value (NPV).

## Methods

This cross-sectional study was conducted at three public primary care clinics in Sepang, Malaysia. The three clinics were purposely chosen because they were managed by the researchers of this study. Through convenience sampling, all patients attending the clinics on Monday, Tuesday and Thursday who attended the clinics during the data collection period (December 2016–April 2017) was screened for their eligibility to be included in this study. The inclusion criteria were adults aged 18 years old and above presenting with a sore throat. We excluded patients with a history of antibiotic administration within the 2 weeks prior to enrolment and pregnant women. All the study subjects were evaluated by a primary care doctor. Demographic and clinical data were collected from the subjects. The researchers documented all variables related to the Centor score. Centor criteria include fever ≥ 38 °C, absence of cough, swollen anterior cervical lymph nodes and tonsillar exudates or swelling. One point is added for each criterion. The Centor scores might range from 0 to 4 (10). Throat swab was collected by the researchers. The study subjects underwent throat swab culture testing to determine GAS infection. Throat swabs were inoculated on a plate of Columbia agar with 5% sheep blood. Any growth isolated were identified by morphology, colony characteristics and biochemical tests. *Streptococcus pyogenes* ATCC 19615 was used as quality control.

The sample size was calculated with single sample proportion sampling formula (11), based on 95% of the confidence interval and significant value at 0.05 and using a previous prevalence of pharyngitis in Thailand (12), considering an estimated 20% incomplete data. The total sample size was 215.

Analyses were conducted using Statistical Package for the Social Sciences (SPSS) software version 22.0 (IBM Corp., Armonk, NY). Categorical variables were presented as whole numbers and percentages. The comparison of categorical variables was done using Pearson’s chi-squared test and Fisher’s exact test as appropriate. A probability of *P* < 0.05 was considered statistically significant. The sensitivity, specificity, PPV, NPV, positive likelihood ratio (LR+) and negative likelihood ratio (LR−) were calculated for Centor scores and clinical manifestations which were statistically significant with GAS pharyngitis. Throat swab culture results were used as reference standards.

## Results

**Figure 1:**
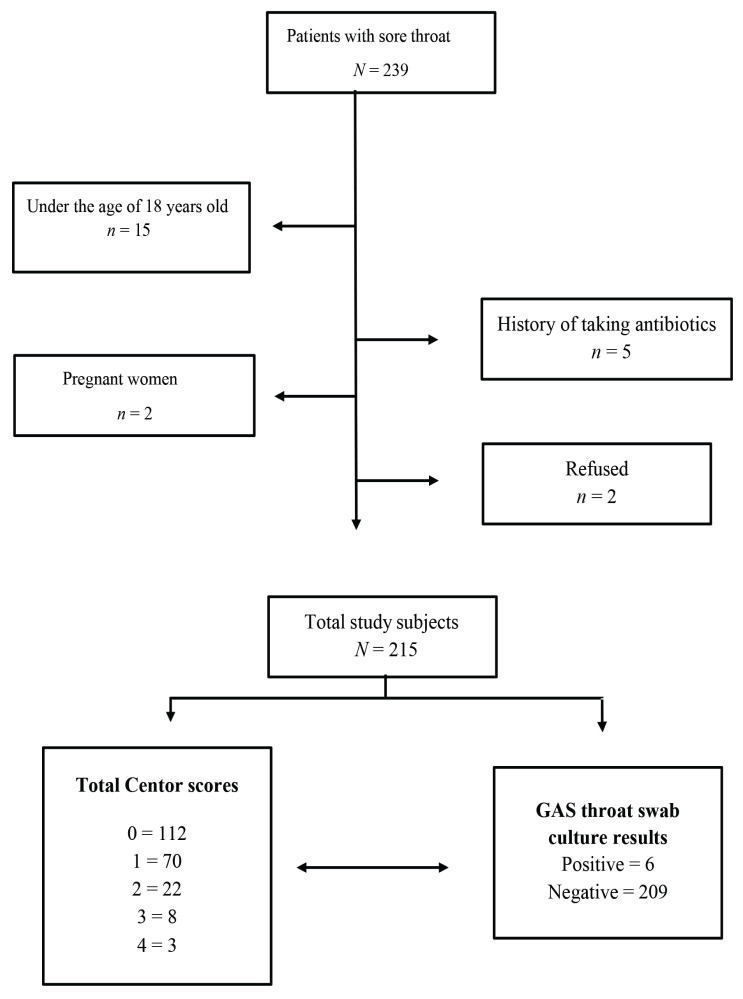
Flowchart of the study

A total of 215 subjects were recruited into this study (Figure 1). Patient demographics and clinical characteristics are summarised in Table 1. The overall mean age (standard deviation [SD]) was 36.43 (SD = 15.7) years old and ranged from 18 years old to 84 years old. All participants were evaluated according to Centor score criteria. The median of Centor score was 0, 52.1% presented with Centor score 0, 32.6% with Centor score 1, 10.2% with Centor score 2, 3.7% with Centor score 3 and 1.4% with Centor score 4. Among 215 throat swab samples, six are isolated GAS.

Table 2 shows the association between the Centor criteria and score with throat swab sample results. There was a significant association between GAS pharyngitis and temperature ≥ 38°C, absence of cough, swollen anterior cervical lymph nodes and tonsillar swelling or exudates. Centor scores of 3 and 4 was significantly associated with GAS pharyngitis.

**Figure 2:**
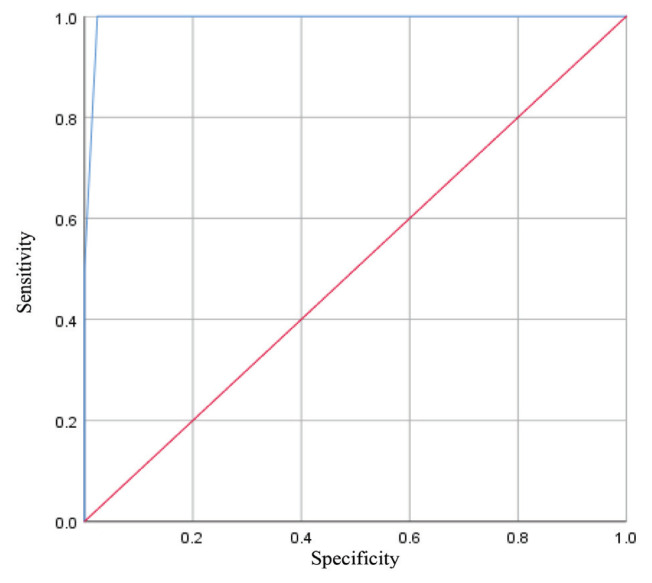
ROC curve of Centor criteria Note: Diagonal segments are produced by ties

Figure 2 shows the receiver operating characteristic (ROC) curve of Centor score. Centor criteria had an area under the curve (AUC) value of 99.4% (95% CI: 98.5, 100%). Table 3 shows the diagnostic test results of Centor criteria scoring, compared to the gold standard of throat swab culture results. Both Centor scores 3 and 4 had a sensitivity of 50% and, specificity of 97.6% and 100%, respectively; PPV 37.5% and 100%, respectively; NPV 98.6% and 98.6%, respectively; LR+ 20.5 and 50; respectively; and LR− 0.5 and 0.5, respectively. The accuracy of Centor score 3 was 96.3%, while the accuracy of Centor score 4 was 98.6%.

## Discussion

In recent years, due to the rise in antimicrobial resistance worldwide, there have been heightened efforts to curb the injudicious use of antibiotics, especially in the outpatient setting. URTIs remain one of the most common presentations amongst primary care patients (2). However, clinical diagnosis of streptococcal pharyngitis has been notoriously unreliable, as clinical signs and symptoms vary, and the severity may range from mild throat discomfort to the classical signs of exudative tonsils with high fever. It is further complicated by the fact that infection due to viruses that do not require antibiotics may be clinically indistinguishable from streptococcal pharyngitis. Throat swab culture or rapid antigen detection testing (RADT) provides a reliable laboratory-based way of diagnosing GAS pharyngitis. However, throat swab culture takes time, and the cost-effectiveness of RADT in low resource settings has yet to be ascertained. To address this shortcoming, clinical scores have been created in an attempt to guide clinicians to the diagnosis of streptococcal pharyngitis and the need to prescribe antibiotics.

Our study found that the proportion of pharyngitis amongst females was higher than amongst males and higher within the 18 years old–28 years old age group, in line with studies performed in Spain and in the United States (13–14). In contrast, a study conducted among Pakistani patients showed that more males suffered from acute pharyngitis than females (15). Our study isolated six GAS pharyngitis (4.6%) from adults with sore throat in the study population. Within the GAS pharyngitis positive group, all (100%) had presence of tonsillar swelling or exudates and swollen anterior cervical lymph nodes, followed by an absence of cough (83.3%), a temperature ≥ 38 °C (66.7%) and rhinorrhoea (16.7%). The proportion of GAS pharyngitis amongst adults in this study was 4.6%. Although almost similar with a study done in Taiwan by Shih et al. (16), this result was low when compared to a Malaysian study carried out among adults (14.2%) and also what has been reported in other parts of the world (15–19). The proportion of GAS pharyngitis among the study population appears to be lower than that of previous studies. This could be explained by the fact that clinics in our study were only public primary care clinics. A national survey reported that most patient encounters in public clinics were for chronic diseases whereas acute complaints such as sore throats were mainly seen in private clinics (20).

Guidelines have recommended the cut-off point for empirical antibiotic treatment to be for cases with Centor score ≥ 3 or with more than three symptoms as there is a higher likelihood that the acute pharyngitis is caused by GAS pharyngitis (21–22). This study found that all the patients with GAS pharyngitis had Centor scores of 3 and 4, and none had Centor scores between 0 and 2. The ROC curves showed that the Centor score has excellent diagnostic value because the curve away from the line of 50% and nearly 100%. The Centor criteria have an AUC value of 99.4 %, meaning that if the Centor score is used to diagnose GAS in 100 patients with sore throat, the correct conclusions will be obtained in 99 of them. AUC value above 90% have excellent interpretation (23). We calculated that Centor scores 3 and 4 to have a sensitivity of 50% and a specificity of between 97.6% and 100% in this study. Centor scores 3 and 4 in this study and previous studies tended to have low sensitivity, but high specificity to rule in diagnosis of GAS pharyngitis (22, 24). The PPV of Centor scores of 3 and 4 in this study were higher than what was recorded by the original study by Centor and other similar studies (10, 22, 25). Even so, PPV in Centor score 3 was not high enough to rule in the diagnosis of GAS pharyngitis. The current recommendation is to perform a throat swab culture or RADT for patients with Centor score 3 (9, 24). The infectious disease society of America (IDSA) recommends antigen testing for adults with Centor score of 2 (26). The NPV of Centor scores of 3 and 4 was similar to that reported in another study (10). The high NPV in this study indicates that Centor score of 4 is an excellent tool to exclude patients without GAS pharyngitis. This value is made more meaningful due to the low prevalence of GAS pharyngitis among adults in the Malaysian population as the score would be able to rule out the diagnosis of GAS pharyngitis when they scored lower.

We studied the association between throat swab cultures and clinical manifestations of patients. Although each clinical criterion was significantly associated with GAS pharyngitis, the specificity (< 95%) and PPV (< 27%) of individual criterion was not high enough to diagnose GAS pharyngitis. A meta-analysis reported that individual Centor criterion had low LR+ (< 2.0) and was not enough to differentiate GAS pharyngitis from non-GAS pharyngitis (27). Although our study showed higher LR+ values, when combined, LR+ values increased greatly to 20.5 for three positive criteria and 50 when all criteria were fulfilled. This finding supports the idea that the Centor criteria in its entirety is a better diagnostic predictor for GAS pharyngitis that any individual signs and symptoms.

This study shows that the Centor score is beneficial in primary care practice. Using Centor scores of 3 and 4 to diagnose GAS pharyngitis would possibly reduce antibiotic prescription rates. However, patients with a Centor score of 3 would need confirmation with a further test.

This study has some limitations: paediatric subjects (under 18 years old) were excluded due to ethical concerns and the need for parental consent. In addition, this study used convenience sampling, which may not accurately represent the current population. We recommend that further studies include all age groups and private and public primary care clinics from different areas to ensure that the whole population of Malaysia is represented.

## Conclusion

Centor scores 3 and 4 have low sensitivity but high specificity, which is associated with GAS pharyngitis. Therefore, Centor scores < 3 may help exclude a diagnosis of GAS pharyngitis, while Centor scores 3 and 4 require further investigation by a throat swab culture or rapid strep throat test to confirm GAS pharyngitis.

## Figures and Tables

**Table 1:** Characteristics of the participants (*N* = 215)

Variables	*n* (%)
Age group (years old)	
Mean age (SD)	36.43 (15.7)
18–28	84 (39.1)
29–39	52 (24.2)
40–50	35 (16.3)
51–60	25 (11.6)
≥ 61	19 (8.8)
Gender	
Male	91 (42.3)
Female	124 (57.7)
Ethnicity	
Malay	135 (62.8)
Indian	65 (30.2)
Chinese	9 (5.1)
Others^a^	4 (1.9)
Smoking status	
Smoker	40 (18.6)
Non-smoker	175 (81.4)
Clinical manifestations	
Cough	196 (91.2)
Rhinorrhea	161 (74.9)
Inflamed and reddish pharynx	63 (29.3)
Fever ≥ 37.5 °C	28 (13)
Fever ≥ 38 °C	15 (7)
Tonsillar swelling or exudates	71 (33)
Swollen anterior cervical lymph	50 (23.3)
Headache	22 (10.2)
Vomiting	8 (3.7)
Centor score	
Score 0	112 (52.1)
Score 1	70 (32.5)
Score 2	22 (10.2)
Score 3	8 (3.7)
Score 4	3 (1.5)
Throat sample results	
GAS	6 (2.8)
Non-GAS	209 (97.2)

Notes:

a Other ethnicities include three Indonesian and one Orang Asli;

*n* = number of respondents; *N* = the sample size of this study; GAS = Group A *Streptococcus*

**Table 2:** Association between Centor criteria and scores with throat swab results (*N* = 215)

Clinical manifestations (*N* = 215)		Throat sample results		*P-*value

		GAS (*n* = 6) *n* (%)	Non-GAS (*n* = 209) *n* (%)	
Temperature 38 °C	Yes	4 (66.7%)	11 (5.3%)	< 0.001*^a^
	No	2 (33.3 %)	198 (94.7%)	
Absence of cough	Yes	5 (83.3%)	14 (6.7%)	< 0.001*^a^
	No	1 (16.7%)	195 (93.3%)	
Swollen anterior cervical lymph nodes	Yes	6 (100%)	44 (21.1%)	< 0.001*^a^
	No	0 (0.0%)	165 (78.9%)	
Tonsillar swelling or exudates	Yes	6 (100%)	62 (29.7%)	0.001*^a^
	No	0 (0.0%)	147 (70.3%)	
Score 0	Yes	0 (0.0%)	112 (53.6%)	0.011*^a^
	No	6 (100%)	97 (46.4%)	
Score 1	Yes	0 (0.0%)	70 (33.5%)	0.181^a^
	No	6 (100%)	139 (66.5%)	
Score 2	Yes	0 (0.0%)	22 (10.5%)	> 0.95 a
	No	6 (100%)	187 (89.5%)	
Score 3	Yes	3 (50%)	5 (2.04%)	0.001*^a^
	No	3 (50%)	204 (97.6)	
Score 4	Yes	3 (50%)	0 (0.0%)	< 0.001*^a^
	No	3 (50%)	209 (100%)	

Notes: *n* = number of respondents;

* Statistical significance at *P* < 0.05;

a Fisher’s exact test;

GAS = Group A *Streptococcus*

**Table 3:** Centor criteria and scores confirmed with throat sample results

Centor criteria and scores	Positive GAS	Sensitivity (%)	Specificity (%)	PPV (%)	NPV (%)	Ac (%)	LR+	LR−
Temperature 38 °C	4	66.7%	94.7%	26.7%	99%	93.9%	12.6	0.4
Absence of cough	5	83.3%	93.3%	26.3%	99.5%	93.0%	12.4	0.2
Swollen anterior cervical lymph nodes	6	100%	78.9%	12.0%	100%	79.5%	4.7	0.1
Tonsillar swelling or exudates	6	100%	70.3%	8.8%	100%	71.2%	3.4	0.1
Centor score 3	3	50%	97.6%	37.5%	98.6%	96.3%	20.5	0.5
Centor score 4	3	50%	100%	100%	98.6%	98.6%	50.0	0.5

Notes: PPV = positive predictive value; NPV = negative predictive value; Ac = accuracy; LR+ = positive likelihood ratio; LR− = negative likelihood ratio; GAS = Group A *Streptococcus*

## References

[1] Anjos LMM, Marcondes MB, Lima MF, Mondelli AL, Okoshi MP (2014). Streptococcal acute pharyngitis. Rev Soc Bras Med Trop.

[2] Teng CL (2014). Antibiotic prescribing for upper respiratory tract infections in the Asia-Pacific region: a brief review. Malays Fam Physician.

[3] Muthanna A, Salim HS, Hamat RA, Shamsuddin NH, Zakariah SZ (2018). Clinical screening tools to diagnose group A streptococcal pharyngotonsillitis in primary care clinics to improve prescribing habits. Malays J Med Sci.

[4] Humair JP, Revaz SA, Bovier P, Stalder H (2006). Management of acute pharyngitis in adults: reliability of rapid streptococcal tests and clinical findings. Arch Intern Med.

[5] Almeman AA, Ibrahim MIM, Rasool S (2014). Cost analysis of medications used in upper respiratory tract infections and prescribing patterns in Universiti Sains Malaysia, Penang, Malaysia. Trop J Pharm Res.

[6] Teng CL, Tong SF, Khoo EM, Lee V, Zailinawati AH, Mimi O (2011). Antibiotics for URTI and UTI prescribing in Malaysian primary care settings. Aust Fam Physician.

[7] Ministry of Health Malaysia (2014). National Antibiotic Guideline 2014.

[8] Andersson DI, Hughes D, Kubicek-Sutherland JZ (2016). Mechanisms and consequences of bacterial resistance to antimicrobial peptides. Drug Resist Updat.

[9] Choby BA (2009). Diagnosis and treatment of streptococcal pharyngitis. Am Fam Physician.

[10] Centor RM, Witherspoon JM, Dalton HP, Brody CE, Link K (1981). The diagnosis of strep throat in adults in the emergency room. Med Decis Making.

[11] Lemeshow S, Hosmer D, Klar W, Lwanga SK (1990). Adequacy of sample size in health studies.

[12] Treebupachatsakul P, Tiengrim S, Thamlikitkul V (2006). Upper respiratory tract infection in Thai adults: prevalence and prediction of bacterial causes, and effectiveness of using clinical practice guidelines. J Med Assoc Thai.

[13] Atlas SJ, McDermott SM, Mannone C, Barry MJ (2005). The role of point of care testing for patients with acute pharyngitis. J Gen Intern Med.

[14] Llor C, Madurell J, Balagué-Corbella M, Gómez M, Cots JM (2011). Impact on antibiotic prescription of rapid antigen detection testing in acute pharyngitis in adults: a randomised clinical trial. Br J Gen Pract.

[15] Rathi SK, Raeefuddin A (2014). Pakistan prevalence survey in acute pharyngitis. J Pak Med Assoc.

[16] Shih CT, Lin CC, Lu CC (2012). Evaluation of a streptococcal pharyngitis score in Southern Taiwan. Pediatr Neonatol.

[17] Foong HBB, Yassim M, Chia YC, Kang BH (1992). Streptococcal pharyngitis in a primary care clinic. Singapore Med J.

[18] Bakare TMO, Schattner P (2010). The usefulness of a clinical ‘scorecard’ in managing patients with sore throat in general practice. Asia Pac Fam Med.

[19] Engel ME, Muhamed B, Whitelaw AC, Musvosvi M, Mayosi BM, Dale JB (2014). Group A streptococcal *emm* type prevalence among symptomatic children in Cape Town and potential vaccine coverage. Pediatr Infect Dis J.

[20] Sivasampu S, Yasmin Farhana AW, Ong SM, Goh PP, Noor Hisham A (2015). National Medical Care Statistics (NMCS) 2014.

[21] Ministry of Health Malaysia Clinical practice guidelines: management of sore throat; 2003.

[22] Aalbers J, O’Brien KK, Chan WS, Falk GA, Teljeur C, Dimitrov BD (2011). Predicting streptococcal pharyngitis in adults in primary care: a systematic review of the diagnostic accuracy of symptoms and signs and validation of the Centor score. BMC Med.

[23] Dahlan MS, Novianty A (2009). Memperoleh nilai area under the curve dengan prosedur receiver operating characteristic. Seri evidence based medicine 5: penelitian diagnostik.

[24] Tanis CD, Abbott AN, Fang FC (2014). Reflexive culture in adolescents and adults with group A streptococcal pharyngitis. Clin Infect Dis.

[25] Mistik S, Gokahmetoglu S, Balci E, Onuk FA (2015). Sore throat in primary care project: a clinical score to diagnose viral sore throat. Fam Pract.

[26] Shulman ST, Bisno AL, Clegg HW, Gerber MA, Kaplan EL, Lee G (2012). Clinical practice guideline for the diagnosis and management of group A streptococcal pharyngitis: 2012 Update by the infectious diseases society of America. Clin Infect Dis.

[27] Thai TN, Dale AP, Ebell MH (2018). Signs and symptoms of group A versus non-group A strep throat: a meta-analysis. Fam Pract.

